# Women’s attitudes towards discontinuation of female genital mutilation in Egypt

**DOI:** 10.5249/jivr.v2i1.33

**Published:** 2010-01

**Authors:** Koustuv Dalal, Stephen Lawoko, Bjarne Jansson

**Affiliations:** ^*a*^Linkopings Universitet, Centre for Medical Technology Assessment & Division of Social Medicine and Public Health, Department of Medical and Health Sciences, Linkopings Universitet, SE-581 83 Linköping, Sweden.; ^*b*^Karolinska Institute,t Depertment of Public Health Sciences, SE 171 76, Stockholm, Sweden.

## Abstract

**Background:**

To examine women's attitude towards discontinuation of female genital mutilation (FGM) in association with their access to information, knowledge of health effects and cultural beliefs concerning FGM in Egypt.

**Methods:**

A cross-sectional study of 9159 women, using data from the household survey in Egypt by Demographic and Health survey 2003. A comprehensive questionnaire covering attitudes towards FGM, demographics, and access to information was used. Chi-square analysis and logistic regression were applied to investigate how demographics, level of education, access to information, knowledge of health consequences and cultural beliefs influence women's attitudes towards FGM.

**Results:**

Among the demographic variables, discontinuation of FGM was independently associated with urban residency and post-secondary education. Moreover, women who were informed by the media, and those who had attended community meetings, church, or mosque where FGM was discussed, as well as women who were aware of the negative health consequences of FGM, were more likely to support discontinuation of FGM. By contrast, women with positive cultural conceptions of FGM were less likely to favor its discontinuation.

**Conclusions:**

Public education and information dissemination aiming to change current cultural notions favoring FGM practice - through community and religious leaders, and radio and television programs - may play an important role in modifying women's attitudes towards FGM. These findings have some implications for intervention and policy.

## Introduction

Female genital mutilation (FGM) has gained increased attention in policy and research over the last decades due to its impact on women’s health, including severe violation of human rights.^1-11^ With an incidence of 2.2 million women per year, global prevalence is increasing rapidly.^12^ More than 135 million women now have experience of FGM.^7^ The problem is mainly confined to Africa and some middle-eastern countries.^5,7^ Also, with increasing migration, the problem is expected to increase in the high-income countries.^13,14,15^

Historically in many cultures practices involving cutting of female genitals have been envisaged. However there is no definitive evidence documenting when or why this ritual started. A group of researchers suggest that FGM might have been initiated in ancient Greece, Rome, Pre-Islamic Arabia and the Tsarist Russian Federation, while others strongly suggest that it might be in ancient Egypt as a sign of distinction.16 FGM has traditionally been called female circumcision and nowadays also known as female genital cutting or FGC.^13,16,17^ The cultural practice of cutting female genitalia for non-medical reason is a harmful phenomenon, especially when the society believes that FGM is the entry point for the girls to become a woman. FGM practice is generally performed to remove all or just part of the external parts of the female genitalia in three different ways.^9,10,18^

I.Sunna Circumcision to remove the prepuce (retractable fold of skin, or hood) and /or the tip of the clitoris. Sunna is an Arabic term, means "tradition".

II. Clitoridectomy to remove the entire clitoris (prepuce and glands) and the removal of the adjacent labia.

III. Pharaonic circumcision (Infibulation) is a clitoridectomy (removal of all or part of the labia minora, the labia majora) with stitching up allowing a small hole to remain open to allow for urine and menstrual blood to flow through.

The age for carrying out FGM is most commonly between four and eight, however might vary from just after birth to sometime during the first pregnancy. In most of the cases FGMs are performed by a senior female member of the family and/or someone in the neighborhood, without an anesthesia and presence of a any trained medical personnel,, due to the poverty and lack of access to medical facilities. The instruments used for FGM could be sharp stone, broken glass, an iron piece, a tin lid, razor blades, knives, scissors, needles or any other sharp object. The instruments are usually not sterilized before or after usage. The older women strongly hold down the girl to prevent her from moving around. After removing her genital area, the child/female is stitched up and her legs are bounded for a few weeks.^9,10,18,19^ Even in some cultures the girls immediately after the performance of FGM, are forced to dance in front of all the members of the community (neighborhood) to prove how womanly they are.

FGM results some serious medical consequences such as infections, abscesses, small benign tumors, hemorrhages, and clitoral cysts. Psychological consequences of FGM are anxiety, horror, post traumatic stress disorders (PTSD) and depression. Long term consequences are chronic problems including difficulties of urination and menstruation, excessive pain with attempts at coital penetration, extreme sensitivity in clitoral remnants, sexual dysfunction and various urinary tract, gynecological and obstetric problem.^9,10,19^

In societies where FGM is practiced, a girl can't be considered an adult/women until she has FGM and hence a girl can't marry without going through FGM. Types of procedure to remove genitalia vary with ethnicities, groups, rural-urban inhabitancy, socio-economic backgrounds and obviously across countries. Therefore, FGM is a ritual that brings about cultural identity and its (FGM) function is to define a group (ethnicity). Therefore, it is believed that removal of such practice eventually would demise the associated culture.^9,10,18,19^

Egypt has the highest incidence of female genital mutilation worldwide.^8,20,21^ Despite the banning of FGM by the Egyptian High Court in 1997,22 recent reports indicate that 97% of Egyptian women have had this experience.^23,24^ The magnitude of the negative physical, and psychological consequences of FGM is well documented, however, being mostly a cultural practice relatively little is known about women’s (the segment of the population mostly affected by this practice) attitudes towards FGM and its determinants. The current study has examined women’s attitude towards discontinuation of female genital mutilation (FGM) in association with their access to information, knowledge of health effects and cultural beliefs concerning FGM in Egypt.

## Methods

**The Egyptian Interim Demographic and Health Survey**

With a population of 79 million, a literacy rate of 58% (68% male, 48% female) and a per capita income of 3,900 USD (PPP adjusted, 2005), Egypt is among the middle-income countries of the world.^25,26^ The Egyptian Interim Demographic and Health Survey of 2003 ^23^ is the seventh demographic and health survey (DHS) undertaken in the country. Like most other DHSs, the EIDHS of 2003 was mainly funded by USAID. The survey was implemented by the Ministry of Health and the National Population Council of Egypt.

**Sample design**

A systematic random sample of 10,000 households was drawn from the main 2003 EIDHS sample (n= 9,159). Households were selected from among the 490 primary sampling units (PSUs) used in the EIDHS of 2003; twenty four additional PSUs were selected from Menya Governorate, and a further 50 from slum areas in greater Cairo. However, the total number of households selected from each governorate is disproportionate to the size of the population in any one. Thus, the EIDHS was not self-weighting at national level. A more detailed description of the sampling procedure is reported elsewhere.^23^

**Participants and study questionnaire**

All women aged 15 to 49 living in or visiting 10,204 households were eligible for interview. A total of 9,159 were interviewed, giving a response rate of 90%. A comprehensive questionnaire covering demographic and health issues was administered. It covered the background of the women and their husbands, empowerment and social status, reproductive history, attitudes towards family planning, maternal health care, antenatal and delivery care, child care and nutrition, child mortality, immunization and health, awareness of and precautions against sexually transmitted diseases, and female genital cutting. For the current paper, the questions concerning female genital mutilation (FGM), demographics and access to information were of primary interest.

**Measures**

**Dependent variable**

Attitudes towards FGM constituted the dependent / outcome variable, and were assessed by asking respondents their opinion on discontinuation of FGM. The response alternatives were “Continue”, “Discontinue” or “Don’t know”. For the current analyses, only responses in the former two categories were included.

**Independent variables**

Data on access to information about FGM were collected by asking respondents whether, over the previous year, they had read about FGM in the printed media, heard of FGM on TV/radio, community meeting/s, gathering in the church/mosque, or had discussed FGM with family or friends.

Beliefs about FGM were assessed by asking respondents whether they agreed/disagreed that FGM was a part of religion, preferred by husbands, that could lead to a girl’s death, could cause problems in pregnancy and delivery, could prevent adultery, or could lessen sexual satisfaction.

The demographic variables included were age, marital status, residential area, education, literacy level, and occupational status.

The survey procedure (e.g. with regard to organization and sampling methods) and instruments received ethical approval from the Institutional Review Board of Opinion Research Corporation (ORC), Macro International Incorporated. In addition, the recommendations of WHO, concerning the assurance of women’s safety while at the same time maximizing disclosure^24^, were followed through the provision of sufficient training and support to the field workers enrolled. The respondents had the opportunity to give informed consent, and were offered a guarantee of privacy.

**Statistical analyses**

Differences in values of the dependent variable (attitudes towards discontinuation of FGM) between participants in different categories of the explanatory variables (i.e. access to information, beliefs about FGM. and demographics) were assessed by chi-square. To estimate the independent associations between the dependent and independent variables, logistic regression was performed. The magnitude and direction of associations were expressed in odds ratios (ORs). For all the statistical analyses, a significance level of p < .05 was employed.

## Results

**Demographic characteristics of women opting for discontinuation of FGM**

Of the total of 9,159 women, 1,658 (18%) opted for discontinuation of FGM (Table 1). A higher proportion of currently married women opted for discontinuation than formally married peers. Urban residents, educated women and literate women more often favored the discontinuation of FGC than rural residents, uneducated/low-educated and illiterate peers, respectively. Finally, a higher proportion of currently employed women chose discontinuation of FGC than their unemployed peers.

**Table:** Table 1: Participants opting for discontinuation of Female Genital Mutilation (FGM) by demographic characteristics

Variables	Number within category a	Opting for discontinuation of FGM number (%)	Adj. Ors 95% C.I.	p-values
Age^ns^				
15-19	375	69 (18.4%)		
20-24	1287	263 (20.4%)		
25-29	1521	315 (20.7%)		
30-34	1527	275 (21.9%)		
35-39	1425	281 (19.7%)		
40-44	1196	241 (20.2%)		
45-49	1143	214 (18.7%)		
Marital status**				
Currently married	7549	1553 (20.6%)		
Formally married	655	105 (16.0%)		
Residential area***				
Urban	3094	1021 (33.0%)	0.50 (0.41 – 0.60)	p<0.001
Rural	5110	637 (12.5%)	1.0	
Education***				
No education	3419	240 (7.0%)	1.0	
Primary education	1410	199 (14.1%)	1.57 (1.16 –2.12)	p<0.001
Secondary education	2734	841 (30.8%)	2.09 (1.23–3.57)	p<0.001
Higher education	641	378 (59.0%)	3.29 (1.83–5.92)	p<0.001
Literacy level***				
Cannot read/cannot read fully	4560	387 (8.5%)		
Can read fully	3638	1270 (34.9%)		
Occupational status***				
Currently working	1797	491 (27.3%)		
Currently not working	6407	1167 (18.2%)		
Total	9159	1658 (18.1%)		

*** Significance levels in chi-square test are denoted by *** p<0.001 and ** p<0.01. Reference categories are denoted by 1.0

However, after adjusting for all other independent variables in this study using a logistic regression only residential area and education were significantly associated with discontinuation of FGM among the demographic variables. Urban residents (Odds ratio = 0.5, C.I. 0.41-0.60; p<0.001) exhibited a higher likelihood of opting for discontinuation of FGM when contrasted with rural peers. In addition, compared to peers having no education, women with primary education (Odds ratio = 1.57, C.I. 1.16-2.12; p<0.001), secondary education (Odds ratio = 2.09, C.I. 1.23-3.57; p<0.001) and higher education (Odds ratio = 3.29, C.I. 1.83-5.92; p<0.001) were more likely to opt for discontinuation of FGM.

**Distribution of attitudes towards discontinuation of FGM by access to information and beliefs concerning FGM**

As illustrated in Table 2, a higher proportion of women who had, during the most recent year, read about FGM in the printed media, heard of FGM on radio or television, or at community meetings or the church/mosque, or discussed FGM with relatives and friends opted for discontinuation of FGM. In addition, a higher proportion of women who believed that FGM can cause death, problems during pregnancy, or make childbirth difficult, chose discontinuation of FGM. A higher percentage of women who did not believe that FGM prevented adultery, is a part of religion, and is preferred by husbands, opted for discontinuation of FGM. Finally, women who believed that FGM lessened sexual satisfaction more often opted for its discontinuation.

**Table:** Table 2: **Participants opting for discontinuation of Female Genital Mutilation (FGM) by access to information on FGM and beliefs about FGM**

Variables	Number within category a	Opting for discontinuation of FGM number (%)	Adj. Ors 95% C.I.	p-values
Read of FGM in media latest year*** (newspaper, magazine)				
Yes	919	491 (53.4%)	0.58 (0.45-0.76)	p<0.001
No	7285	1167 (16.0%)	1.0	
Heard of FGM on radio latest year***				
Yes	1767	476 (26.9%)		
No	6437	1182 (18.4%)		
Heard of FGM on TV latest year***				
Yes	6893	1555 (22.6%)	0.45 (0.33-0.60)	p<0.001
No	1311	103 (7.9%)	1.0	
Heard of FGM on Community meeting latest year***				
Yes	165	68 (41.2%)	0.43 (0.27-0.70)	p<0.01
No	8039	1590 (19.8%)	1.0	
Heard of FGM on mosque /church latest year***				
Yez	348	174 (70.3%)	0.55 (0.39-0.78)	p<0.01
No	7856	1484 (18.9%)	1.0	
Discussed FGM latest year*** (with friends/ relatives)				
Yes	4811	1061 (22.1%)		
No	3392	596 (17.6%)		
FGM can lead to girls death***				
Agree	2230	1101 (49.4%)	0.33 (0.28-0.40)	p<0.001
Disagree	5150	459 (8.9%)	1.0	
FGM causes problems in getting pregnant***				
Agree	731	365 (49.9%)	0.72 ( 0.54-0.96)	p<0.001
Disagree	5922	1005 (17.0%)	1.0	
FGM makes child birth more difficult***				
Agree	589	261 (44.3%)	0.45 ( 0.33-0.62)	p<0.001
Disagree	5724	978 (17.1%)	1.0	
FGM prevents adultery***				
Agree	3799	291 (7.7%)	4.00 (3.26-4.88)	p<0.001
Disagree	2661	1033 (38.8%)	1.0	
FGM lessens sexual satisfaction***				
Agree	2608	899 (34.5%)	0.58 (0.47-0.72)	p<0.001
Disagree	3106	393 (12.7%)	1.0	
FGM important part of religion***				
Agree	5858	399 (6.8%)	12.11 (9.99-14.68)	p<0.001
Disagree>	1584	1110 (70.1%)	1.0	
Husbands prefers circumcised woman***				
Agree	5397	341 (6.3%)	5.80 (4.74-7.09)	p<0.001
Disagree	1648	933 (56.6%)	1.0	

*** Chi-square test: Statistically significantly associated with discontinuation of FGM at p<0.001. Reference categories are denoted by 1.0

After adjusting for other independent variables, using logistic regression analysis, the results indicated-- a higher likelihood of opting for discontinuation of FGM among women who, during the most recent year have had access to information about FGM via the printed media (Odds ratio = 0.58, C.I. 0.45-0.76; p<0.001), Television (Odds ratio = 0.45, C.I. 0.33-0.60; p<0.001), community meeting/s (Odds ratio = 0.43, C.I. 0.27-0.70; p<0.01), or mosque/church gatherings (Odds ratio = 0.55, C.I. 0.39-0.78; p<0.01) when contrasted with peers lacking access to information about FGM. Further, women who believed that FGM could lead to a girl’s death (Odds ratio = 0.33, C.I. 0.28-0.40; p<0.001), cause problems during pregnancy (Odds ratio = 0.72, C.I. 0.54-0.96; p<0.01) or make childbirth difficult (Odds ratio = 0.45, C.I. 0.33-0.62; p<0.001) showed a higher likelihood of opting for discontinuation of FGM than peers with no such beliefs. Women who believed that FGM prevented adultery (Odds ratio = 4.00, C.I. 3.26-4.88; p<0.001), is a part of religion (Odds ratio = 12.11, C.I. 9.99-14.68; p<0.001), and is preferred by the husbands (Odds ratio = 5.80, C.I. 4.74-7.09; p<0.001) were less likely to opt for discontinuation of FGM than their respective peers with no such beliefs. Finally, women who believed that FGM lessened sexual satisfaction (Odds ratio = 0.58, C.I. 0.47-0.72; p<0.001) were more likely to opt for discontinuation than peers with no such belief.

## Discussion

Previous studies have demonstrated that almost all Egyptian women (97%) have been subjected to FGM.^8^ In the current study, up to 82% of the women supported the continuation of FGM. Our findings suggest that tolerant attitudes towards FGM, in combination with a social gradient may play a significant role in the persistence of FGM among the Egyptian women. It is therefore important to understand how factors associated with attitudes towards FGM.

In our sample, the attitude of the women towards FGM was associated with their social status. Women that living in the urban areas, having a higher level of education/literacy, and those who were working, were more likely to support discontinuation of FGM. However, only the residential-area and education variables remained significantly associated with discontinuation of FGM in the multivariate analysis. Furthermore, access to information was independently associated with a less tolerant attitude towards continuation of FGM. It seems that educationally empowered women, are more exposed to the controversy surrounding the FGM.^8,16^

These findings underscore the importance of public education, urbanization and information dissemination in changing women’s attitudes to FGM. In the most affected countries/regions, women’s organizations and advocates argue that such interventions may be more effective in changing women’s attitudes against FGM practice than pure legislations against FGM.^5,28^ Earlier studies have also recommended that development and implementation of legislation against FGM, solely, is not an effective way to reduce its prevalence. They suggest adequate educational and awareness raising campaigns are necessary to inform the general public about risks of this ritual.^8,9,10,29^

Women who were aware of the negative health consequences of FGM (e.g. mortality, difficulties in pregnancy, and sexual dissatisfaction) were found to favor discontinuation of FGM. Educational interventions that emphasize the negative consequences of FGM can help to lower favorable attitude among Egyptian women. Brief counseling on the unsafe consequences of FGM by the healthcare provide can also help to inform women about the health-related consequences of this practice.

In this study, women with culturally-based beliefs about FGM (e.g. that FGM prevents adultery, is an important part of religion, is preferred by the husbands) were more likely to justify FGM. These are women who seem to believe that FGM has a positive function for the institutions of marriage, family, and religion. At the same time our findings revealed that women who had heard about FGM at meetings in the community, mosque or church were less likely to opt for continuation of FGM. These findings point to the important role that the institution of religion can play in informing its believer about the health compromising affects of FGM. Community leaders and women’ health advocates should also take advantage of the community gathering and events to disseminate educational information about the negative consequences of FGM to their members.

To the best of our knowledge, current study is the first to examine the attitude of the women regarding discontinuation of FGM in Egypt. Existing empirical findings on this topic, for the most part, have focused on the attitudes of the health service providers.^29^ Findings of the current study not only support provision and dissemination of adequate education about FGM but adds to the existing knowledge by pointing to the role of decision-makers and leaders in the community and religious leaders as the channels of intervention to modify cultural beliefs about FGM.

Our results are limited to providing only associational relationship between the main variables. The cross-sectional design of this study does not allow for assigning causality. In other words, the direction of the relationship between attending community meetings, going to a mosque, or a church, and reporting a positive attitude toward discontinuation of FGM can't be assessed by the current data. It is plausible that women in favor of discontinuation of FGM are more likely to seek information. Thus, studies with a longitudinal design are needed to confirm potential causal links.

In sum, our study has pointed to some of the important implications for interventions and policy developments with regard to FGM. Structural improvement (e.g. public education) and changes to specific cultural beliefs, channeled through community and religious leaders, radio and television, may be of paramount importance in modifying women’s attitudes towards FGM.
